# Prognostic Utility of Optical Coherence Tomography for Visual Outcome After Extended Endoscopic Endonasal Surgery for Adult Craniopharyngiomas

**DOI:** 10.3389/fonc.2021.764582

**Published:** 2022-01-06

**Authors:** Ning Qiao, Chuzhong Li, Jing Xu, Guofo Ma, Jie Kang, Lu Jin, Lei Cao, Chunhui Liu, Yazhuo Zhang, Songbai Gui

**Affiliations:** ^1^ Department of Neurosurgery, Beijing Tiantan Hospital, Capital Medical University, Beijing, China; ^2^ Department of Cell Biology, Beijing Neurosurgical Institute, Capital Medical University, Beijing, China; ^3^ Department of Ophthalmology, Beijing Tiantan Hospital, Capital Medical University, Beijing, China

**Keywords:** optical coherence tomography, prognosis factors, visual outcomes, extended endoscopic endonasal surgery, craniopharyngiomas

## Abstract

**Introduction:**

Owing to the close vicinity of the optic chiasma, visual dysfunction is known as one of the most common surgical indications and postoperative complications in adult patients with craniopharyngiomas, probably leading to poor quality of life. Historically, very few consistent predictive factors associated with the visual outcome are identified, which may not be helpful for patient counseling and preoperative decision making. Recently, optical coherence tomography (OCT) serving as a novel high-resolution imaging technique can assess the retinal morphology by measuring the circumpapillary retinal nerve fiber layer (cpRNFL) and macular ganglion cell complex thickness. However, few studies have examined the prognostic utility of OCT parameters for visual outcome after surgery for craniopharyngiomas. This study aims to use the largest series to evaluate the association between OCT parameters and visual outcome after extended endoscopic endonasal surgery (EEES) for primary craniopharyngiomas in adults.

**Material and Methods:**

From October 2018 to October 2020, one hundred and seventy eyes in 88 adult patients with newly confirmed craniopharyngiomas were retrospectively reviewed and pertinent prognostic factors were analyzed.

**Results:**

Gross total resection was performed in 82 (93.2%) patients. The median postoperative follow-up time was 10.9 months. Multiple logistic regression analysis showed that increased temporal cpRNFL thickness was associated with higher odds of visual acuity (VA) improvement and maintenance (OR = 1.070; 95% CI, 1.005–1.140; p = 0.035), and greater inferior cpRNFL thickness was significantly associated with visual field (VF) improvement and maintenance (OR = 1.034; 95% CI, 1.001–1.068; p = 0.046). Furthermore, tight adhesion between optic nerves and craniopharyngiomas was demonstrated as an independent adverse factor for either postoperative VA or VF (p = 0.048, p = 0.030, respectively). The ROC results further verified the robustness of the prediction model either in VA (AUC = 0.843; 95% CI, 0.734–0.952; p < 0.001) or VF (AUC = 0.849; 95% CI, 0.741–0.958; p < 0.001).

**Conclusion:**

Preoperative OCT can effectively predict visual outcome after EEES for adult craniopharyngiomas. It can also serve as a reliable alternative to evaluate preoperative visual field defects, especially for patients with lower compliance. Tight adhesion was confirmed as an independent risk factor for postoperative visual outcome. The OCT-based multivariable prediction models developed in the present study may contribute to patient counseling on visual prognosis.

## Introduction

Craniopharyngiomas are rare brain tumors originating from any point along with the pituitary–hypothalamic axis, accounting for 1.2%–4.6% of all intracranial tumors (1, 2). Because of the close vicinity of optic chiasma, visual deterioration is known as a common complication following surgery for craniopharyngiomas (3–7). Prognostic factors related to postoperative visual outcome, including age (8–10), symptoms duration (11), tumor size and volume (12), preoperative visual function (10), and optic atrophy (13), have been studied extensively, but results are not consistent.

Retrograde axonal degeneration caused by chronic optic nerve compression secondary to craniopharyngiomas often leads to thinner circumpapillary retinal nerve fiber layer (cpRNFL) and macular ganglion cell complex (mGCC), thus leading to irreversible visual dysfunction (14). Hence, visual recovery largely relies on timely removal of optic nerve compression and the amount of viable axions (14, 15). Optical coherence tomography (OCT) can serve as a non-invasive *in vivo* method to quantitatively and objectively measure cpRNFL thickness and mGCC parameters (14, 16). The clinical efficiency of OCT as a predictor of visual recovery after surgery for pituitary adenomas, meningiomas, or pediatric craniopharyngiomas has already been verified (13, 14, 16–21). Differing from pituitary tumors and meningioma, craniopharyngiomas often directly adhere to optic nerves, with a higher risk of postoperative visual deterioration. Compared with pediatric craniopharyngiomas, adult craniopharyngiomas more frequently cause visual impairment before surgery (5, 22). Therefore, investigating reliable predictive indicators of postoperative visual outcome may be beneficial for patients counseling on visual prognosis. However, there is limited evidence on the prognostic utility of OCT for visual outcome after surgery for adult craniopharyngiomas (16, 18).

This is the first study to systematically evaluate the association between OCT parameters and visual outcome after the extended endoscopic endonasal surgery (EEES) for adult craniopharyngiomas.

## Materials and Methods

### Patient Population

**Figure 1:**
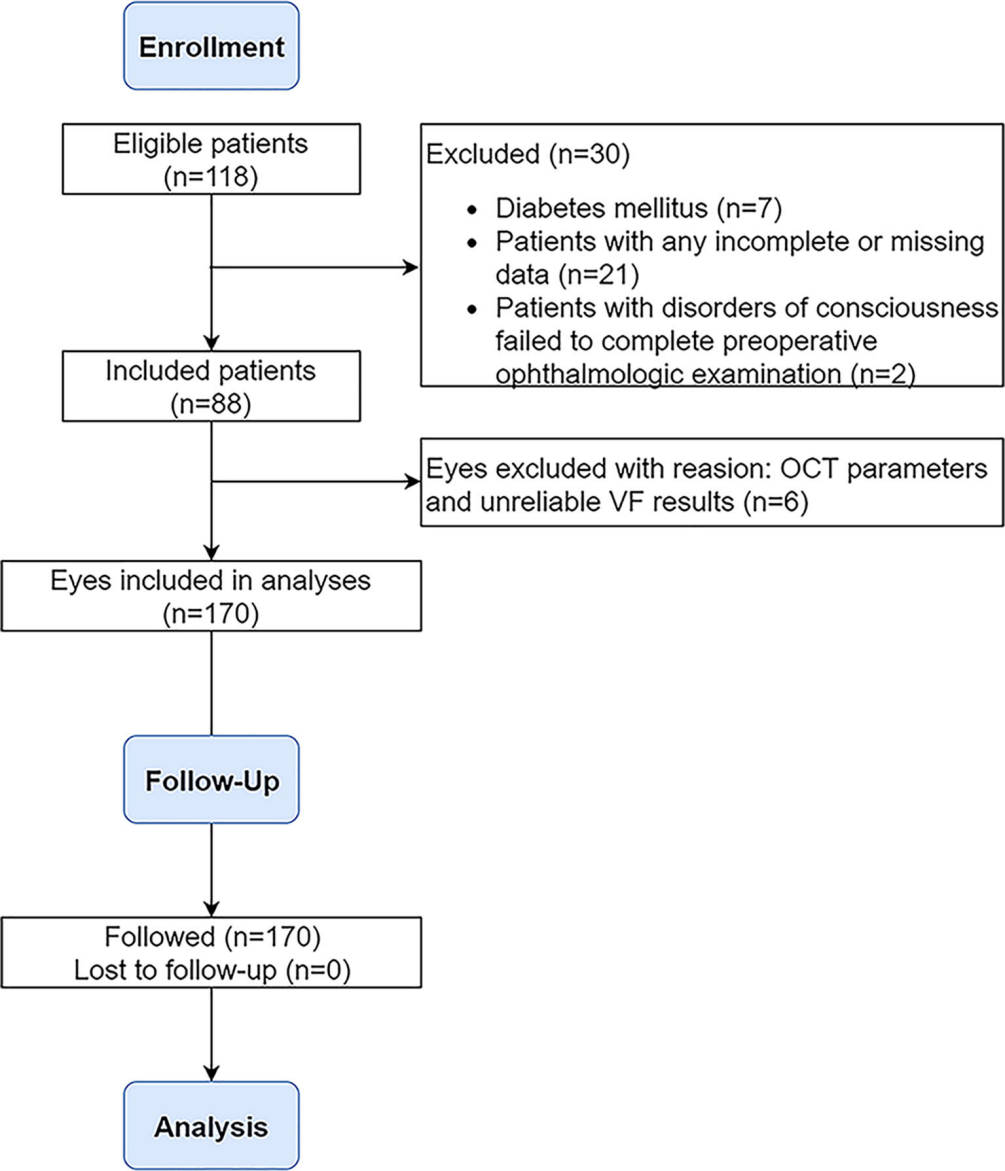
Flowchart demonstrating the inclusion/exclusion criteria used in the selection process. OCT, optical coherence tomography; VF, visual field.

From October 2018 to October 2020, a total of 118 adult patients underwent EEES for primary craniopharyngiomas at Beijing Tiantan Hospital of Capital Medical University. Inclusion criteria were as follows: (1) adult patients aged ≥18 years, (2) newly confirmed diagnosis of craniopharyngioma, (3) computed tomography (CT), magnetic resonance imaging (MRI), and ophthalmologic tests before and after surgery. The exclusion criteria were (1) past medical history of treatment including radiotherapy and surgery, (2) any ophthalmic condition other than compressive optic neuropathy caused by craniopharyngiomas, (3) any medical illness (including glaucoma, diabetes mellitus) known to affect optic apparatus, (4) ineligible OCT parameters, (5) unreliable visual field (VF) and best-corrected visual acuity (BCVA) testing (fixation losses more than 20%, false-negative error more than 20%, and false-positive error more than 20%), (6) myopia greater than –6.00 diopters, (7) and papilledema on fundoscopy. Consequently, 88 (74.6%) of 118 patients with primary craniopharyngiomas were retrospectively analyzed in this study. The flowchart for study inclusion and exclusion is described in **Figure 1**. All participants signed an informed consent form. The study was approved by the ethics committee of Beijing Tiantan Hospital of Capital Medical University.

### Radiological Evaluations

The MRI examinations were performed preoperatively and at 3 and 9 months after surgery. Subsequent MRI scans were executed annually. Gross total removal (GTR) was defined as the resection without visual residual enhancing tumor according to postoperative MRI (5). Tumor recurrence during follow-up was defined as the development of a pathological lesion on MRI that had not previously been observed or the regrowth of tumor residuals (5). Tumor volume was calculated by using the following formula (23): volume = 4/3π (a/2 × b/2 × c/2) (where a, b, and c represent the diameters in the three dimensions).

### Visual Evaluation and Definition

The ophthalmologic tests were performed preoperatively and at least 3 months after surgery. The BCVA was evaluated using a logarithmic visual acuity chart and then converted to the logarithm of the minimum angle of resolution (logMAR) for analysis. The VF examinations, including mean deviation (MD), pattern standard deviation (PSD), and visual field index (VFI), were performed using the Humphrey field analyzer (24-2 SITA-fast program, Carl Zeiss Meditec, Dublin, California, USA). OCT measurements, including cpRNFL thickness and mGCC parameters, were conducted using spectral-domain OCT (Optovue, Freemont CA, USA). OCT parameters were also analyzed based on a decade of age: ≤20, 21–30, 31–40, 41–50, 51–60, and 61–70 years (24, 25). For the analysis, improvement or worsening in BCVA (normal ≥ 1.0) was defined as a change of greater than 0.1 in LogMAR visual acuity (26). The VF improvement or worsening was defined as a change of MD [normal ≥ -2 decibels (dB)] greater than -3 dB (27).

### Spectral-Domain Optical Coherence Tomography

Subjects underwent spectral-domain optical coherence tomography (SD-OCT) scanning without pupillary dilation using Avanti RTVue XR (Optovue, Fremont, California, USA) by experienced examiners on the same day as the ophthalmic evaluation. This equipment with an axial scan speed of 100 kHz using an 840-nm-wavelength laser has a resolution of 5.3 mm axially and 18 mm laterally. Three consecutive scans were performed on each eye. The scanning protocol for peripapillary RNFL thickness was acquired using the optic nerve head map, with a scanning range covering centered on the optic disc and covering a circle 3.45 mm in diameter. The GCC thickness was obtained using the GCC scanning protocol, which generates the data through the scans of a square grid (7 mm × 7 mm) on the central macula centered 1 mm temporal to the fovea and covered. Criteria for acceptable images included signal intensity level greater than 7 of 10, signal strength index ≥40. The normal RNFL and GCC thickness was defined as within the 95% percentile of age-, sex-, and race-matched normative values obtained from the manufacturer’s database.

### Surgical Procedures

All extended endoscopic endonasal approaches were performed by one surgeon (SG). Firstly, a right middle turbinectomy, nasoseptal flap harvesting, posterior septal resection, and an enough opening of the sphenoid sinus were performed. Subsequently, the tuberculum sellae is removed using a Kerrison rongeur and a high-speed drill, and the bony removal was extended anteriorly toward the planum sphenoidale and laterally to the medial optic-carotid recess bilaterally. When the dura mater was opened, the arachnoid membrane was sharply dissected, and the tumor was exposed between the upper surface of the pituitary gland and the optic chiasm (28). If the pituitary stalk was confirmed to suffer from obvious tumor invasion, it was sacrificed (29). After assessment of the pituitary stalk, the tumor was debulked adequately. When necessary, sharp separation of the tumor from neurovascular structures like optic nerves, optic chiasma, and hypothalamus was performed. After the removal of the tumor, skull base reconstruction was performed according to our earlier literature (30).

### Classification of Adhesion

**Figure 2:**
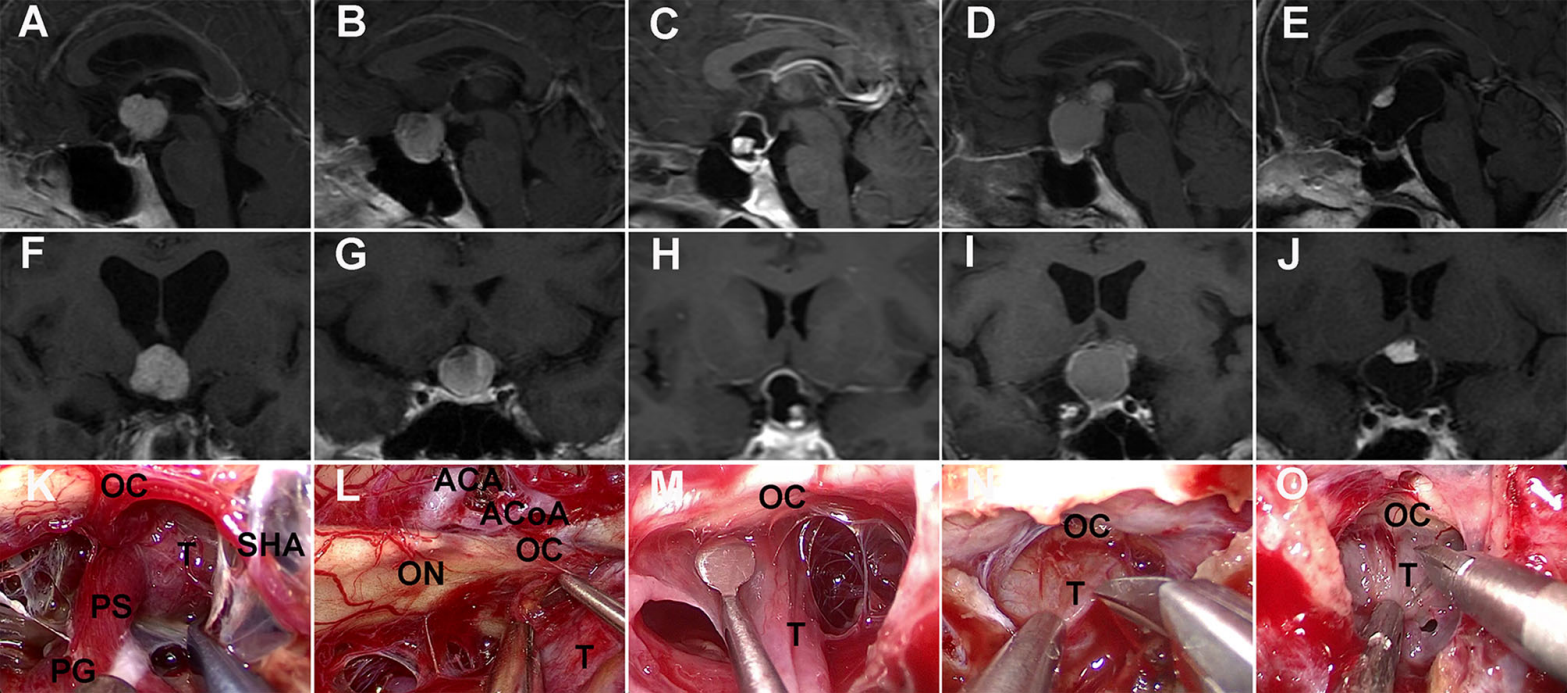
Adhesion strength between craniopharyngioma and optic nerves was intraoperatively evaluated. **(K, L, M)** No or loose adhesion. Contrast-enhanced T1-weighted MRI scans **(A, F)** showing an intrinsic third ventricular solid tumor compressing forward the optic chiasm. Intraoperative view **(K)** revealing that the proximal part of the pituitary stalk extending from the gland could be identified as intact and at a normal size before tumor resection. Preoperative MRI scans **(B, C, G, H)** showing a sellar-suprasellar/suprasellar cystic-solid tumor stretching upward the optic chiasm. Surgical view **(L, M)** showing that the tumor can be easily separated from the optic nerves by dissector. **(N, O)** Tight adhesion. Preoperative MRI scans **(D, E, I, J)** showing sellar-suprasellar cystic-solid tumors displacing the optic chiasm. Intraoperative video-captured photographs **(N, O)** showing tight adhesion between the tumor and the optic apparatus needing sharp dissection using scissors. OC, optic chiasma; ON, optic nerve; T, tumor; PG, pituitary gland; PS, pituitary stalk; SHA, superior hypophysial artery; ACA, anterior cerebral artery; ACoA, anterior communicating artery.

Compared to pituitary adenomas, craniopharyngiomas posed challenges mainly owing to their tendency to adhere to vital neurovascular structures, such as optic nerves and optic chiasma (31), with a higher risk of postoperative visual deterioration. The adhesion strength between optic apparatus and the tumor was classified into two categories according to intraoperative findings by the surgeon: (1) no or loose adhesion if the tumor can be easily separated from the optic apparatus by gentle blunt dissection using dissectors or (2) tight adhesion if the separation of the tumor required sharp dissection using scissors (**Figure 2**).

### Statistical Analysis

We performed all statistical analyses with SPSS statistics software version 23 (IBM Corp). The data were presented as the mean ± standard deviation (SD) or median (with interquartile range (IQR)) for normally distributed and non-normally distributed samples, respectively. Differences between the pre- and postoperative visual outcomes were assessed by using the Wilcoxon signed-rank test. Spearman’s rank correlation coefficients were used to evaluate the relationship between OCT and VF parameters. The prognostic factors for visual outcome were analyzed by binary logistic regression. Variables were selected into the multivariate analysis according to a statistically significant association in univariate analysis (p < 0.05) or previous studies and professional knowledge (32). Independent predictors in multivariate analysis and other variables selected by referring to previous studies and professional knowledge were used to establish the multivariable prediction models. Receiver operating characteristic (ROC) curves were used to determine the performance of the prediction model. The area under the curve (AUC) with 95% confidence interval (CI) and the associated p-value were both calculated. p < 0.05 was considered statistically significant.

## Results

### Patient Characteristics

The present cohort included 37 (42.0%) male patients, and the mean age was 44.0 years old (range, 19–68 years). The most common preoperative symptom was visual impairment (78 patients; 88.6%), and the mean duration of such symptom was 6.2 months (range, 1–24 months). The median tumor volume was 6.5 cm^3^ (IQR, 3.4-14.0 cm^3^). The clinicoradiological data of 88 patients are shown in **Table 1**.

**Table 1:** Clinicoradiological characteristics of the 88 enrolled patients.

Parameters	Values, n (%)
Total number	88
Sex	
Male	37 (42)
Female	51 (58)
Age, y	44.0 ± 13.1
Preoperative manifestations	
Visual disturbance	78 (89)
Menstrual disorder/impaired sexual function	70 (80)
Headache	58 (66)
Fatigue	43 (49)
Polyuria/polydipsia	33 (38)
Preoperative visual acuity	
Normal	23 (26)
Abnormal	65 (74)
Preoperative visual field	
No defect	10 (11)
Defect	78 (89)
Size of tumor	
Volume (cm)	6.5 (IQR,3.4-14.0)
Characteristics of tumor	
Solid	12 (14)
Cystic	31 (35)
Solid and cystic	
Cystic component >50%	29 (33)
Solid component >50%	16 (18)
With hydrocephalus	33 (37.5)
With calcification	49 (55.7)
Preoperative cpRNFL parameters (μm)	
Average thickness	97.05 ± 13.17
Superior quadrant	124.66 ± 20.24
Inferior quadrant	121.72 ± 19.36
Nasal quadrant	70.53 ± 13.96
Temporal quadrant	70.28 ± 12.42
Preoperative mGCC parameters (μm)	
Inner average	91.68 ± 9.15
Superior	91.00 ± 9.34
Inferior	92.36 ± 9.49
GCC FLV (%)	3.19 ± 2.94
GCC GLV (%)	6.49 ± 5.88

Values are presented as number (%), mean ± standard deviation, or median [with interquartile range (IQR)]. cpRNFL, circumpapillary retinal nerve fiber layer; mGCL, macular ganglion cell layer; GLV, global loss volume; FLV, focal loss volume.

### Preoperative Visual Function

One hundred and twenty-three eyes (72.4%) had VA impairment preoperatively. The median BCVA was 0.2 logMAR (IQR, 0 to 0.5). VF defects occurred in one hundred and forty-nine eyes (87.6%). MD, PSD, and VFI on VF testing were -9.3 (IQR, -14.8 to -4.9), 7.7 (IQR, 3.5 -11.4), and 77.5 (IQR, 56.5-90), respectively. The mean global RNFL thickness was 97.05 ± 13.17 μm. It was 121.72 ± 19.36 μm in the inferior quadrant, 124.66 ± 20.24 μm in the superior quadrant, 70.53 ± 13.96 μm in the nasal quadrant, and 70.28 ± 12.42 μm in the temporal quadrant, respectively. Inner average, superior, and inferior mGCC thicknesses were 91.68 ± 9.15 μm, 91.00 ± 9.34 μm, and 92.36 ± 9.49 μm, respectively (**Table 1**). The associations between the mGCC parameters, cpRNFL thickness parameters, and VF parameters in the 170 eyes are shown in **Table 2**. mGCC parameters significantly correlated with MD, PSD, and VFI. All cpRNFL thickness parameters were significantly associated with MD except for the superior quadrant, PSD except for the nasal quadrant, and VFI except for the inferior and nasal quadrant, respectively.

**Table 2:** Relationship between GCC parameters, RNFL thickness parameters, and preoperative visual field parameters.

Variable	MD		PSD		VFI	
	r	p value	r	p value	r	p value
cpRNFL parameters						
Average thickness	0.226	0.003*	-0.342	<0.001*	0.245	0.001*
Superior quadrant	0.266	<0.001*	-0.347	<0.001*	0.288	<0.001*
Inferior quadrant	0.140	0.069	-0.225	0.003*	0.124	0.106
Nasal quadrant	-0.039	0.611	-0.086	0.266	0.003	0.971
Temporal quadrant	0.327	<0.001*	-0.356	<0.001*	0.325	<0.001*
mGCC parameters						
Inner average	0.264	0.001*	-0.293	<0.001*	0.239	0.020*
Superior	0.288	<0.001*	-0.318	<0.001*	0.267	<0.001*
Inferior	0.235	0.002*	-0.259	<0.001*	0.203	0.008*
Focal loss volume	-0.342	<0.001*	0.367	<0.001*	-0.356	<0.001*
Global loss volume	-0.332	<0.001*	0.334	<0.001*	-0.317	<0.001*

cpRNFL, circumpapillary retinal nerve fiber layer; mGCL, macular ganglion cell layer; MD, mean deviation; VFI, visual field index; PSD, pattern standard deviation.

The asterisk indicates statistical significance, p < 0.05.

### Overall Surgical Results

GTR was performed in 82 (93.2%) patients. Of the six cases with residual tumors, three were observed without further treatment, and three received gamma-knife radiosurgeries postoperatively without causing new visual defects. Tight adhesion was observed in 31 (35.2%) patients. Adamantinomatous craniopharyngiomas were confirmed in 67 (76.1%) patients. After a median follow-up duration of 10.9 months, recurrence occurred in 2 (2.3%) patients. Of these patients, one did radiotherapy, and the other was observed without adjuvant therapy. There was no new visual impairment occurred in these two patients.

### Postoperative Visual Outcome

The follow-up time was 10.9 (IQR, 7.2–16.2) months. Among 123 eyes with preoperative VA impairment, VA improved in 78.0% but worsened in 4.9% postoperatively. Five (10.6%) of the 47 eyes with normal preoperative VA had postoperative VA deterioration. Of the 149 eyes with preoperative VF impairment, 83 (55.7%) experienced improved or normalized VF, with no change in 58 (38.9%), and 8 (5.4%) experienced deterioration after surgery. Eighteen (85.7%) of 21 eyes with normal preoperative VF showed no change, and 2 (9.5%) experienced worsening. The median BCVA after surgery was 0.1 logMAR (IQR, 0 to 0.2), which was significantly lower than the preoperative 0.2 logMAR (IQR, 0 to 0.5) (p <0.001). The MD (IQR) showed a significant improvement from -9.3 (IQR, -14.8 to -4.9) preoperatively to -5.3 (IQR, -9.9 to -2.5) postoperatively (p < 0.001).The mean global RNFL thickness after surgery was 86.99 ± 13.99 μm. It was 112.81 ± 18.37 μm in the inferior quadrant, 112.02 ± 20.41 μm in the superior quadrant, 60.46 ± 14.03 μm in the nasal quadrant, and 62.67 ± 12.26 μm in the temporal quadrant, respectively. Inner average, superior, and inferior mGCC thicknesses were 87.19 ± 10.26 μm, 86.33 ± 10.85 μm, and 88.08 ± 10.12 μm, respectively. While overall visual function showed significant improvement following surgery, all postoperative OCT parameters mentioned above significantly decreased compared with preoperative data (each p < 0.001).

### Prognostic Factors for Visual Prognosis

**Figure 3:**
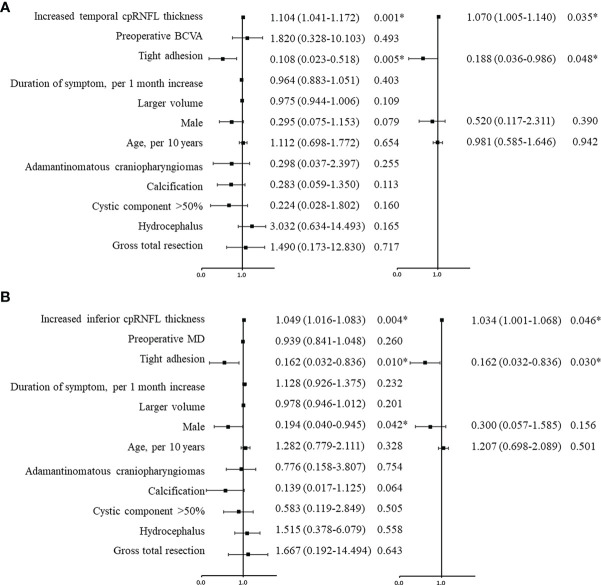
Univariate and multivariate logistic regression analyses were used to evaluate the predictive factors for visual prognosis following surgery for craniopharyngiomas. The black squares indicate the OR values, error bars represent 95% CIs, and *p < 0.05. According to the analysis, increased temporal **(A)** and inferior **(B)** cpRNFL were favorable factors for postoperative visual acuity and visual field, respectively. Tight adhesion was an adverse factor for visual recovery. cpRNF, circumpapillary retinal nerve fiber layer; BCVA, best corrected visual acuity; MD, mean deviation.

**Figure 4:**
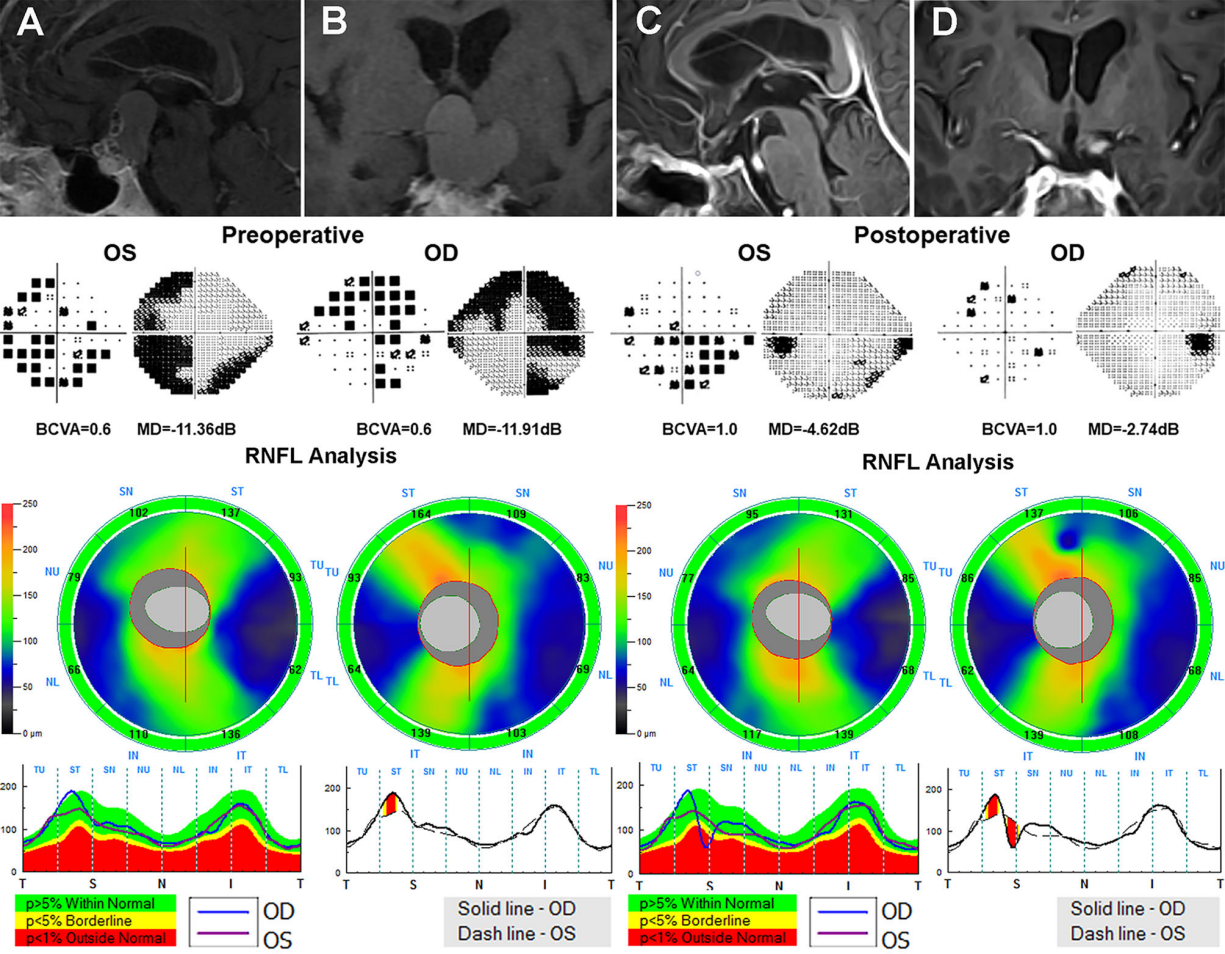
A 59-year-old male patient who underwent the EEES for craniopharyngiomas was examined preoperatively and 3 months after surgery. Contrast-enhanced T1-weighted MRI scans **(A, B)** suggested a suprasellar cystic-solid lesion compressing downward the optic chiasma. Preoperative Humphrey VF test showed mainly temporal VF defects in both eyes. Preoperative OCT suggested normal cpRNFL thickness in both eyes. After total resection of the tumor **(C, D)**, the optic nerves were sufficiently decompressed, the VA and VF in both eyes dramatically improved after surgery. EEES, extended endoscopic endonasal surgery; OCT, optical coherence tomography; cpRNF, circumpapillary retinal nerve fiber layer; BCVA, best-corrected visual acuity; MD, mean deviation; VA, visual acuity; VF, visual field.

**Figure 5:**
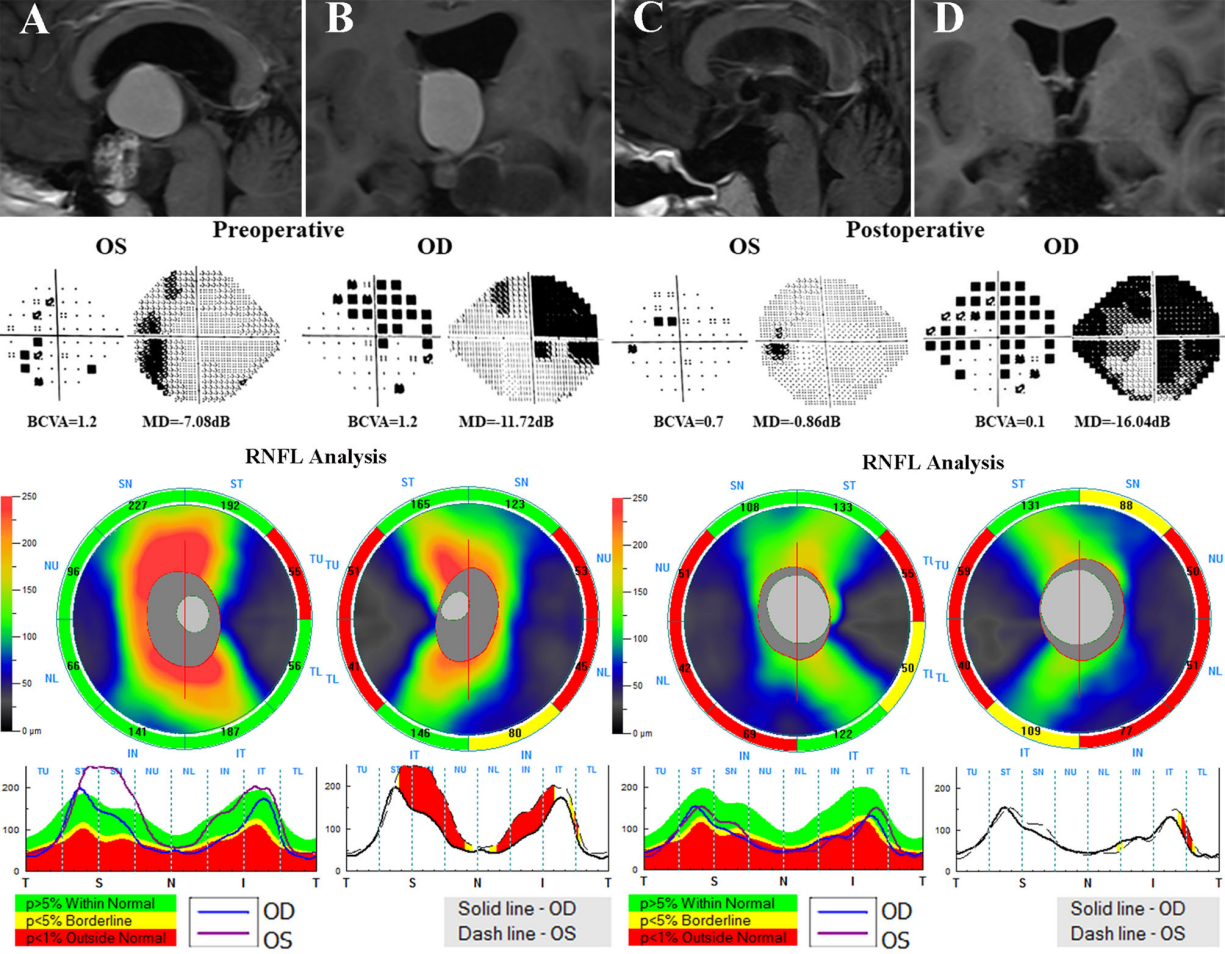
A 21-year-old male who underwent endoscopic surgery for craniopharyngiomas was evaluated before and after surgery. Preoperative MRI scans **(A, B)** reveals a suprasellar cystic-solid tumor involving the third ventricle compressing the optic chiasma. Preoperative visual field examination suggested bitemporal VF defects. Preoperative OCT showed decreased temporal cpRNFL thickness in both eyes and inferior cpRNFL thinning in the right eye. After surgery **(C, D)**, the deterioration of VA in both eyes and VF in the right eye were observed. OCT, optical coherence tomography; cpRNF, circumpapillary retinal nerve fiber layer; BCVA, best-corrected visual acuity; MD, mean deviation; VA, visual acuity; VF, visual field.

Univariate logistic regression analysis for visual improvement and maintenance by OCT parameters are summarized in **Table 3**, and increased temporal (p = 0.001) and inferior cpRNFL thickness (p = 0.004) proved to be independent prognostic factors. Clinicoradiological factors were also assessed, and the univariate analysis results revealed that tight adhesion and gender were associated significantly with postoperative visual outcome. In the multivariate analysis, increased temporal (OR, 1.070; 95% confidence interval [CI], 1.005–1.140; p = 0.035) and inferior cpRNFL thickness (OR, 1.034; 95% CI, 1.001–1.068; p = 0.046) proved to be independent favorable factors for VA (**Figure 3A**) and VF (**Figure 3B**) improvement and maintenance after surgery, respectively (**Figures 4**, **5**). Moreover, tight adhesion was confirmed as an independent risk factor for VA (OR, 0.188; 95% CI, 0.036–0.986; p = 0.048) or VF (OR, 0.162; 95% CI, 0.032–0.836; p = 0.030) after surgery for craniopharyngiomas.

**Table 3:** Univariate logistic regression for visual improvement and maintenance by OCT parameters.

Variable	VA improvement and maintenance		VF improvement and maintenance	
	OR (95% CI)	p value	OR (95% CI)	p value
cpRNFL thickness (μm)				
Average	1.036 (0.990–1.083)	0.127	1.012 (0.965–1.061)	0.630
Superior	1.012 (0.982–1.043)	0.440	0.990 (0.960–1.021)	0.509
Inferior	1.019 (0.989–1.051)	0.216	1.049 (1.016–1.083)	0.004*
Nasal	1.020 (0.976–1.066)	0.375	1.018 (0.972–1.066)	0.444
Temporal	1.104 (1.041–1.172)	0.001*	1.027 (0.975–1.082)	0.313
mGCC parameters (μm)				
Inner average	1.056 (0.988–1.128)	0.109	1.049 (0.979–1.124)	0.175
Superior	1.056 (0.991–1.126)	0.091	1.041 (0.974–1.112)	0.235
Inferior	1.046 (0.983–1.113)	0.152	1.049 (0.983–1.119)	0.146
GCC FLV (%)	0.860 (0.723–1.022)	0.087	0.958 (0.781–1.175)	0.681
GCC GLV (%)	0.933 (0.855–1.019)	0.122	0.950 (0.865–1.044)	0.290

OCT, optical coherence tomography; VA, visual acuity; VF, visual field; BCVA, best-corrected visual acuity; MD, mean deviation; cpRNFL, circumpapillary retinal nerve fiber layer; mGCL, macular ganglion cell layer; GLV, global loss volume; FLV, focal loss volume.

The asterisk indicates statistical significance, p < 0.05.

**Figure 6:**
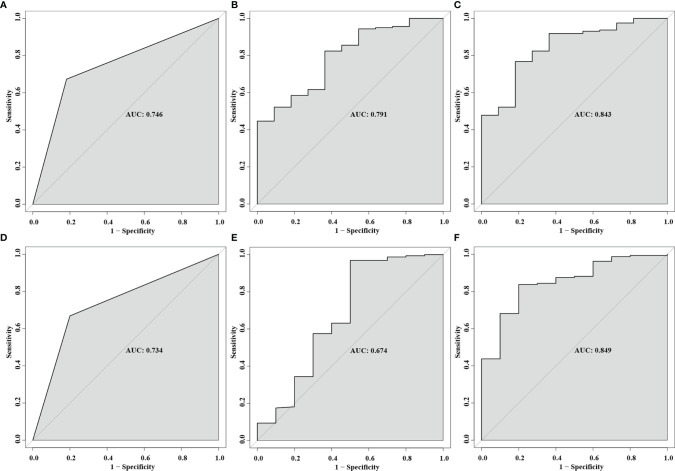
Receiver operating characteristic (ROC) curves for the predictor of multivariable prediction models developed for postoperative VA: tight adhesion **(A)**, increased temporal cpRNFL **(B)**, and the combination of all predictors **(C)**. ROC curves for the predictor of multivariable prediction models developed for postoperative VF: tight adhesion **(D)**, increased inferior cpRNFL **(E)**, and the combination of all predictors **(F)**.

As for predictors of postoperative VA, the AUC was 0.791 (95% CI, 0.667–0.914; p = 0.001) for temporal cpRNFL thickness and 0.746 (95% CI, 0.605–0.887; p = 0.007) for tight adherence, respectively. In terms of predictive factors of postoperative VF, the AUC with was 0.674 (95% CI, 0.459–0.890; p = 0.065) for inferior cpRNFL thickness and 0.734 (95% CI, 0.583–0.886; p = 0.013) for tight adherence, respectively. Multivariable prediction models developed for postoperative VA and VF recovery and maintenance, including age, gender, cpRNFL thickness, and adhesion strength, showed AUC of 0.843 (95% CI, 0.734–0.952; p < 0.001) and 0.849 (95% CI, 0.741–0.958; p < 0.001), respectively (**Figure 6**).

## Discussion

Retrograde axonal degeneration resulting from chronic compression of optic chiasma can result in cpRNFL and mGCC thinning, consequently leading to irreversible visual impairment (14). OCT allows quick, non-invasive, *in vivo* cross-sectional imaging of the retinal layers, acting as an important tool for objective quantification of cpRNFL and mGCC (18). There is increasing evidence suggesting that preoperative OCT parameters can serve as excellent prognostic indicators of visual outcome after surgery for parasellar lesions, such as meningioma, pituitary adenoma, and craniopharyngioma (14, 18, 33–35). Among these tumors, craniopharyngiomas often directly adhere to the undersurface of optic nerves or chiasm, with a higher risk of postoperative visual deterioration (36). Hence, it is helpful for patients with craniopharyngiomas to establish reliable multivariable prediction models to give them good counsel on visual prognosis. In this paper, the authors present the largest series to date to systematically analyze the prognostic utility of OCT parameters for visual outcome after EEES for adult craniopharyngiomas.

In keeping with the results reported in the previous studies (14, 18, 33–35), our research showed that preoperative OCT parameters can effectively predict visual outcome after EEES for adult craniopharyngiomas. Interestingly, increased inferior cpRNFL thickness was significantly associated with higher odds of VF recovery and maintenance (p = 0.046) in the present study, which was consistent with the results reported in the earlier literature (16, 37). Compression of retinal ganglion cell axons at the chiasm usually first cause VF defects because it preferentially damages the ventral fibers from the inferonasal and inferotemporal retina (38). Axonal shrinkage caused by such longstanding compression can bring about the inferior cpRNFL thinning, therefore resulting in irreversible VF defects after decompression. In addition, greater temporal cpRNFL thickness was confirmed as a significant favorable factor for VA recovery and maintenance (p = 0.035) in our study, which was similar to the findings by Kawaguchi and colleagues (26). Within the retina, the axons from the macula project to the disc at the temporal poles, forming the papillomacular bundle responsible for central visual acuity (16, 38). The authors discuss that the potential mechanisms might be that the temporal cpRNFL thinning caused by chiasmal compression secondary to craniopharyngiomas might affect the papillomacular bundle, thus leading to the decreased VA recovery following surgery. In consideration of different tissue types mentioned above, namely, pituitary adenomas (16), meningiomas (38), and craniopharyngioma (17, 20), which belonged to slow-growing benign tumors (WHO grade 1), preoperative cpRNFL thinning might be mainly due to longstanding mechanical compression of the optic apparatus and/or supporting vascular structures, thereby leading to visual impairment. The most noteworthy characteristic of visual impairment with chiasmal compression was that decompression could contribute to immediate visual improvement. Such rapid recovery was not observed in other forms of optic nerve injury (38). However, in terms of functional pituitary adenomas and malignant tumors in the sellar region, the issue of whether disease-related specific factors may affect the OCT parameters and visual outcome or not still required further advanced research.

Noticeably, Yoo et al. (35) argued that the validity of the mGCC thickness measured by SD-OCT in predicting the postoperative visual outcome of parasellar tumors was superior to cpRNFL thickness. Some patients with mild VF defects were reported to have normal cpRNFL thickness, and the benefits of cpRNFL analysis were limited because of axonal overlap around the optic nerve head which did not allow to properly evaluate the topographic arrangement of the retinal ganglion cells (39–41). At present, the mGCC thickness can be measured separately by using SD-OCT. In a series of 79 consecutive patients, Yoo and colleagues (35) pointed out that the mGCC thickness can serve as an excellent predictor of visual recovery after chiasmal decompression. Furthermore, Ohkubo and colleagues (34) declared that preoperative mGCC parameters measured by SD-OCT, particularly focal loss volume (FLV), were shown to be a reliable predictor of visual outcome following surgical decompression of chiasmal compression. Our findings also suggested that the superonasal quadrant mGCC thicknesses (p = 0.091) and FLV (p = 0.087) showed statistical tendencies for VA recovery and maintenance in univariate analysis, although they were not statistically significant. This may be explained by the potential impact of the selection bias on results obtained from the present series.

Interestingly, in our findings, cpRNFL and mGCC thickness showed a significant decrease after surgery while postoperative visual function improved overall, which was consistent with results reported in the series by Lee (37) and Chung (42). An ongoing decline in RNFL thickness without recovery over 6 months following surgery was reported by Lee et al. In another study, Chung and colleagues observed that the RNFL thickness continuously decreased over 12 to 36 months although the visual field recovered. This might be explained by the nerve edema to a certain extent, which potentially contributed to a false increase in OCT results at the preoperative testing, followed by a normalization of OCT parameters after surgery. Based on this, we supported that excluding cases with papilledema on fundoscopy from the analysis probably made it possible to control the possible confounding effect in our study. Another potential mechanism was that permanent ischemia to the outermost layer may lead to gradual thinning although satisfactory decompression of the optic nerve fiber (42). However, the discrepancy between anatomic change and functional recovery still necessitated advanced research in the future.

Moreover, the present study pointed out that tight adhesion between craniopharyngiomas and optic nerves was demonstrated as an independent risk factor for visual outcome following EEES for primary craniopharyngiomas, which was similar to our previous results (36). In the current series, tumors with tight adherence were observed in 31 (35.2%) of all patients. Poor visual outcomes were more likely to happen in patients with tight adherence (35.5%) compared with the rest of patients (1.8%). This could be explained by tumor adherence to the undersurface of optic nerves or chiasm, which can predispose the optical apparatus to mechanical and ischemic injury during the tumor resection.

Operative trauma can be a confounder to postoperative visual outcome (14). Compared with transcranial approaches (5), the extended endoscopic endonasal approach can provide a close-up view with better visualization of optic nerves and facilitate a lower visual deterioration after surgery (4, 6, 7), probably because there was less surgical trauma. Besides, this potential limitation was overcome by using the data of only one neurosurgeon (SG). In addition, in our series, the mean follow-up time was 12.0 months (range, 3–28 months), which is longer than the period reported in the series by Danesh-Meyer that the majority of visual recovery was inclined to happen within the first 6–10 weeks (19). Considering the biological characteristic of craniopharyngiomas, the degree of the adhesion strength between optic nerves and the tumor was evaluated according to intraoperative findings and included in multivariate analysis, which made it possible to control the possible confounding effect.

In our study, the advancing age and gender failed to be predictors of postoperative visual outcome, which was inconsistent with the results of previous studies (8, 13, 43), presumably because of the selection and referral bias of the study population. However, considering the age-related changes and sexual differences in OCT parameters, these factors still needed to be included in multivariate analysis when using cpRNFL thickness to make clinical prediction models (24, 25, 44, 45). Overall, clinical prediction models established in the present study, incorporating age, gender, cpRNFL thickness, and adhesion strength, suggested moderate discriminative abilities of VA (AUC = 0.843) or VF (AUC = 0.849) recovery and maintenance. It may be helpful to patient counseling on visual prognosis.

Our study also showed a statistically significant association between OCT parameters and MD/PSD/VFI, which was in line with the findings reported in the series by Ohkubo (34) and Chung (42). That means OCT parameters can excellently act as an alternative to assess preoperative visual field defects resulting from chronic chiasmal compression, particularly for patients with lower compliance.

### Limitation

The single-center setting and a retrospective study design have the potential to introduce selection bias, and our results required external validation in the future. In addition, the comparison of the extent of long-term visual recovery after surgery among the patients with normal and thin RNFL thickness was limited by the present follow-up time, and ophthalmologic examinations should continue to be termly performed after surgery in the future.

## Conclusion

Preoperative OCT proved to have an independent predictive value in visual outcome after extended endoscopic endonasal surgery for adult craniopharyngiomas. It can also serve as a reliable alternative to evaluate preoperative visual field defects, especially for patients with lower compliance. Tight adhesion was also a strong predictor of postoperative visual outcome. The OCT-based multivariable prediction model developed in the current study may be beneficial to patient counseling on visual prognosis.

## Data Availability Statement

The raw data supporting the conclusions of this article will be made available by the authors, without undue reservation.

## Ethics Statement

The studies involving human participants were reviewed and approved by the ethics committee of Beijing Tiantan Hospital of Capital Medical University. The patients/participants provided their written informed consent to participate in this study.

## Author Contributions

All authors take responsibility for the integrity and the accuracy of this manuscript. Study concept and design: NQ and SG. Draft of the manuscript: NQ, CZL and JX. Acquisition of data: NQ, CHL, GM, JX, JK, and LJ. Statistical analysis: NQ and LC. Edit: NQ. Supervision: CHL and YZ. Revision: NQ and SG. All authors contributed to the article and approved the submitted version.

## Funding

This study was supported by the Beijing Municipal Science & Technology Commission (Z19110700660000) and Beijing Hospitals Authority Clinical Medicine Development of Special Funding Support (XMLX202108).

## Conflict of Interest

The authors declare that the research was conducted in the absence of any commercial or financial relationships that could be construed as a potential conflict of interest.

## Publisher’s Note

All claims expressed in this article are solely those of the authors and do not necessarily represent those of their affiliated organizations, or those of the publisher, the editors and the reviewers. Any product that may be evaluated in this article, or claim that may be made by its manufacturer, is not guaranteed or endorsed by the publisher.
